# The motivational beliefs and attitudes about writing of international students enrolled in online academic English classes during the COVID-19 pandemic

**DOI:** 10.3389/fpsyg.2023.1232664

**Published:** 2024-06-13

**Authors:** Penelope Collins, Michael S. Leo, Maryam Eslami, Michael Hebert, Julian Levine, Jerry Won Lee

**Affiliations:** ^1^School of Education, University of California, Irvine, Irvine, CA, United States; ^2^English Department, University of California, Irvine, Irvine, CA, United States

**Keywords:** writing, composition, motivation, affect, efficacy, goal orientation, L2 learners, international students

## Abstract

Despite the growing attention to motivation, less is known about international students’ motivational beliefs and attitudes about academic writing. In this study, we aimed to explore the motivational factors influencing international students’ performance in academic English classes at a large public research university in the western United States. Specifically, we examined students’ self-efficacy, goal orientation, beliefs, and affect for writing, along with their malleability, and their contributions to academic achievement in academic English writing classes. The sample comprised 97 students, predominantly from China, enrolled in online academic English courses. Exploratory factor analysis tended to extract more complex models of the motivational constructs than principal component analysis. Students’ self-efficacy and enjoyment of writing significantly increased from the beginning to the end of the 10-week term, suggesting motivational factors’ malleability. Hierarchical linear modeling revealed that students’ self-efficacy at the beginning of the term positively predicted their final grades. However, logistic mixed modeling revealed that students who held stronger beliefs about writing as a means of exploring and expressing ideas had lower odds of passing. Our findings contribute to the understanding of international students’ motivation in academic English settings in higher education and offers potential pedagogical interventions to enhance their academic success.

## Introduction

In the last decade, the number of international students enrolled in American universities has increased by 26%, to over 900,000 students (Institute of International Education, 2022). Key reasons for studying abroad include receiving a high-quality education at a prestigious institution, increased future earnings, and gaining global cultural capital (Kim et al., 2018). However, many international students face both acculturative and academic challenges in their educational pursuits (Wu et al., 2015; Heng, 2018), with their academic writing skills in their second language (L2), English, being an oft-cited barrier to success (Andrade, 2006; Sherry et al., 2010). Many universities offer basic English as a Second Language (ESL) or academic English writing classes to provide students with the necessary skills and confidence to succeed in their academic writing tasks (Flowerdew and Peacock, 2001; Ferris and Hedgcock, 2004; Bauer and Picciotto, 2013). However, factors beyond English language proficiency, such as international students’ motivation, self-efficacy, beliefs, and affect also play pivotal roles in English language writing and, by extension, their academic achievement (Phakiti et al., 2013; Zheng et al., 2018).

In addition to globalizing the student body, universities have increased online course offerings over the past decade (Xu and Xu, 2019). The pandemic’s shift to emergency remote teaching and the subsequent proliferation of post-pandemic online instruction has metamorphosed the educational landscape, making it accessible to a broader spectrum of learners (Johinke et al., 2023). Although online instruction has some advantages, such as greater access and flexibility for students and improved progress to degree completion (Xu and Xu, 2019; Fischer et al., 2020; Martin et al., 2022), it poses new challenges for students, such as a more considerable need for self-regulation skills and intrinsic motivation (Broadbent and Poon, 2015; Xu and Xu, 2019). More specifically, motivational factors such as self-efficacy, goal orientation, beliefs about learning, and affect contribute to students’ course engagement and self-regulation, which, in turn, promote superior academic outcomes in their online courses (Broadbent and Poon, 2015; Alemayehu and Chen, 2021). There is some evidence to suggest that international students with greater self-efficacy, positive beliefs, and affinity for English language learning have enjoyed greater success in their online courses (e.g., Zheng et al., 2018; Wang and Zhan, 2020). In contrast, disengagement from the curriculum, low self-efficacy, and anxiety about English language learning have been found to impede international students’ success in online writing classes in English during the pandemic (Lin and Nguyen, 2021).

Whereas motivation is a multifaceted construct that plays an essential role in writing achievement and academic success (MacArthur et al., 2016; Ling et al., 2021), its contributions to L2 writing performance among international students needs to be better understood. On the one hand, international students often experience pressure to succeed academically, which, coupled with the stress of adjusting to a new cultural environment, may exacerbate generalized stress and anxiety about writing in English (Pappamihiel, 2001; Yeh and Inose, 2003). Further, international students may experience stigma associated with remedial, academic English courses, which can lead to negative attitudes and lower motivation (Moss et al., 2014). On the other hand, international students’ academic histories before studying abroad may bolster their motivational beliefs and affect for writing in English. Unlike domestic students in remedial writing classes who show pervasive motivational problems for writing due to their history of academic struggles (MacArthur et al., 2016), international students may have more productive motivational beliefs and attitudes for writing and learning due to their strong records of academic success in their home countries and primary language. Students’ motivation, beliefs, and affect for writing in their first language (L1) have been found to transfer to their L2 (Saeli and Cheng, 2019; Zhu et al., 2022). Consequently, while international students may need the support of academic English classes to better prepare them for the writing demands of undergraduate schooling in English, their past academic achievements and language learning experiences may lead them to exhibit more productive self-efficacy, beliefs, and attitudes toward writing than their domestic peers. Thus, there are mixed and sometimes contradictory accounts of international students’ motivation for writing in English. Further, many studies have addressed in-person instruction (Saeli and Cheng, 2019; Zhu et al., 2022), yet less is known about the roles these factors play for international students when academic English classes are delivered online.

### Academic English writing and motivation for international students

Writing effectively in an academic context is a complex, multifaceted process that requires domain knowledge, language proficiency, and an understanding of rhetoric and genre conventions (Scarcella, 2003; Bazerman et al., 2017). For international students, academic writing in English is particularly challenging (Robertson et al., 2000). Writing in English requires not only linguistic knowledge of English (Flower and Hayes, 1981; Graham and Perin, 2007) but also a nuanced understanding of the cultural and rhetorical norms and expectations of academic writing in English (Silva, 1993; Zamel, 1995; Connor, 2004; Wang and Zhan, 2020), some of which vary across disciplines. Adjusting to a new educational system can be particularly challenging because the expectations and norms of academic writing in English-speaking countries may differ significantly from those in the students’ home countries (Zamel, 1997). Thus, it is important to study the motivation, beliefs, and attitudes of international students in academic English classes, as these can affect their ability to develop writing skills in English. Indeed, international students’ English language learning experiences may shape their motivation, self-efficacy, attitudes, and beliefs about writing, which may drive their efforts, persistence, and success in mastering writing skills.

Motivation plays an important role in contemporary models of writing (Hayes, 1996; Graham, 2018). Motivation is critical in the learning process, driving students’ engagement, persistence, effort, and academic performance (Eccles and Wigfield, 2002; Pintrich and Schunk, 2002). Motivation is a complex, multidimensional construct shaped by individual characteristics and contextual factors, and when combined with self-regulatory processes, guides student choice, effort, persistence, and achievement (Pintrich and Zusho, 2007). These dimensions reflect a range of interrelated factors, such as their confidence, personal goals, beliefs, values, and emotions (Troia et al., 2012; Conradi et al., 2014; MacArthur et al., 2016; Camacho et al., 2021). Researchers have adopted four dimensions of motivation to explore writing development: self-efficacy, goal orientation, beliefs about writing, and affect (Troia et al., 2012; MacArthur et al., 2016).

The motivational construct that has arguably received the greatest attention from writing researchers is self-efficacy (Camacho et al., 2021). Self-efficacy, a concept derived from Bandura’s (1977) social cognitive theory, refers to a person’s judgment of their ability to complete a specific task or reach a particular goal successfully. That is, students with high levels of self-efficacy have high expectations that they will complete a task successfully, leading them to be more willing to engage or persist in challenging learning tasks (Eccles and Wigfield, 2002; Pintrich and Schunk, 2002). In writing, self-efficacy describes students’ confidence in their writing skills to accomplish writing tasks (Pajares, 2003). However, it is less clear whether self-efficacy for writing can be considered a unitary construct (e.g., Zimmerman and Bandura, 1994; MacArthur et al., 2016) or if it involves distinct factors for basic grammar skills and advanced composition skills (Pajares, 2007). Although single-factor models of self-efficacy have been extracted with undergraduate students who may have greater mastery of the conventions of writing and the writing process (MacArthur et al., 2016; Ling et al., 2021), the two-factor model was supported among K-12 students still learning the conventions, discourse structures and modes of inquiry involved in writing (Pajares, 2007). Because international students enrolled in academic English writing programs may still be developing these skills in English, it is unclear whether self-efficacy for English academic writing can best be characterized as a single- or dual-factor construct.

Self-efficacy is a robust contributor to undergraduate students’ writing engagement, persistence, and achievement (Zimmerman and Bandura, 1994). Among multilingual students writing in English, whether in English as a foreign language (EFL) contexts or as international students, self-efficacy often shares a positive relationship with English writing achievement (Phakiti et al., 2013; Chea and Shumow, 2017; Sabti et al., 2019). However, some studies of international students have found self-efficacy to be unrelated to English writing achievement (Wilby, 2022) or correlated with the use of vocabulary and conventions in English writing but not with compositional skills, such as the quality of argumentation, ideation (Ling et al., 2021). Further, most examinations of the role of self-efficacy in international students’ writing performance have been conducted when the instruction has been delivered in person rather than in online learning environments (e.g., Phakiti et al., 2013; Chea and Shumow, 2017; Sabti et al., 2019). Because self-efficacy contributes to student engagement and general achievement in online learning environments (Alemayehu and Chen, 2021; Teng, 2021), self-efficacy may have a more robust role in international students’ achievement in academic English courses delivered online.

In addition to self-efficacy, researchers have applied achievement goal theory to explain writing achievement (e.g., Troia et al., 2012; MacArthur et al., 2016). Goal orientation refers to the situated reasons why an individual engages in a specific task (Pajares and Cheong, 2003; Kaplan and Maehr, 2007). These goals may be adaptive or maladaptive and have been thought to reflect mastery or performance orientations (Dweck, 1986; Ames, 1992). Mastery-oriented goals emphasize the development of competence and learning (Dweck, 1986; Elliot and McGregor, 2001; Pajares and Cheong, 2003). Mastery orientation is considered adaptive, as students who are concerned with developing their skills show greater persistence and seek out challenges to gain skills (Ames, 1992; Pintrich, 2000). In contrast, two types of performance orientations are considered less adaptive, as students are driven to perform for the sake of being judged favorably (*performance-approach orientation*) or to avoid negative evaluation (*performance-avoidance orientation*; Elliot and McGregor, 2001). Students with performance-approach goal orientations seek to appear competent for recognition or higher grades, whereas those with performance-avoidance goals seek to avoid displaying incompetence or failure (Elliot and Church, 1997; Pintrich, 2000). Students with performance goals may experience heightened anxiety levels and avoid challenging tasks that might expose their perceived shortcomings (Dweck, 1986). Further, performance orientations are often associated with weaker persistence and exerting less cognitive effort (Pajares and Cheong, 2003). Although goal orientation is often thought of as three distinct factors (Elliot and Church, 1997; MacArthur et al., 2016), mastery goals have been found to share moderate to strong correlations with performance-approach goals (Troia et al., 2013; MacArthur et al., 2016; Chea and Shumow, 2017; Sabti et al., 2019).

The relationships between undergraduates’ writing achievement and their goal orientations are mixed in the literature. For example, MacArthur et al. (2016) found that undergraduates’ mastery and performance-approach goals were uncorrelated with most writing measures, but that avoidance goals negatively impacted writing performance. Farsani et al. (2014) found goal orientation was unrelated to writing achievement in English among EFL undergraduates, whereas other researchers found mastery goals, but not approach or avoidance goals, were correlated with EFL students’ writing achievement in English (Chea and Shumow, 2017; Zerey and Müjdeci, 2023). Further, Wilby (2022) reported that mastery and performance-approach goals, but not avoidance goals, were correlated with international students’ essay scores. Thus, the relationship between goal orientation and writing achievement in English among international students remains unclear.

Self-efficacy and goal orientation may guide the degree of effort students exert in writing tasks, but their beliefs about what constitutes good writing may inform how they direct their efforts. Beliefs about writing span a broad spectrum of ideas and assumptions that students hold about the nature of writing and what constitutes good writing (White and Bruning, 2005). These beliefs encompass students’ perceptions of writing to explore and express ideas and the importance of proper grammar and conventions (MacArthur et al., 2016; Ling et al., 2021). Studies have shown that these beliefs significantly sway students’ motivation, writing performance, and eagerness to participate in writing tasks (Bruning and Horn, 2000; Pajares, 2003). These beliefs can either boost or obstruct an individual’s writing ability (Flower and Hayes, 1981), as students whose beliefs about writing focus on meaning show greater cognitive engagement while writing (Schraw, 2000; White and Bruning, 2005). Examinations of students’ implicit and explicit beliefs about what constitutes good writing have typically yielded two-factor models, with beliefs about the roles of ideas and beliefs about writing conventions loading onto two distinct factors (MacArthur et al., 2016; Ling et al., 2021).

Although students’ beliefs about writing may guide how they engage in the writing process, their relationship with writing quality is less clear. The relationship between beliefs about content among undergraduate students varies depending on the writing task. For example, beliefs about the importance of ideas and content have been positively correlated with the HEIghten Critical Thinking and Written Communication assessment, which evaluates students’ critical thinking, analytic, and synthesis skills (Ling et al., 2021). However, content beliefs shared negative correlations with the Accuplacer writing test that focuses on sentence construction and logic and standardized measures of writing fluency (MacArthur et al., 2016). Finally, content beliefs were unrelated to students’ persuasive essay writing (MacArthur et al., 2016). The relationship between beliefs and writing achievement in English may be more complex for international students writing in their L2, as their beliefs about what constitutes quality writing in their L1 may not match the rhetorical and argumentative conventions of academic writing in English (Connor, 2004; Heng, 2018). For example, the critical thinking, analysis, evidence-based arguments, and citation conventions expected in American universities may be unfamiliar to international students due to cultural differences in instruction (Wu, 2015; Heng, 2018). Therefore, the mismatch between their prior learning experiences and the conventions of academic writing in English may lead international students to hold strong beliefs about the content of writing that may be unhelpful in American universities. Indeed, whereas EFL students’ beliefs about writing were positively correlated with self-efficacy, mastery goals, and performance-approach goals, their beliefs about writing were unrelated to the English writing scores (Zerey and Müjdeci, 2023).

In addition to self-efficacy for writing, goal orientation and beliefs about writing may be shaped by affective factors. Affect pertains to the emotional experiences and feelings associated with writing tasks, such as anxiety, frustration, enjoyment, and satisfaction (Pekrun, 2006). Students’ affective responses to writing influence their choices and engagement and moderate their performance in writing tasks (Pekrun et al., 2002; Graham, 2018). Whereas positive emotions such as enjoyment and satisfaction can amplify students’ effort and persistence in writing tasks, negative affect can interfere with students’ thinking and engagement in writing (MacArthur et al., 2016; Graham, 2018; Ling et al., 2021). For example, high writing anxiety levels can result in avoidance behaviors, diminished effort, and subpar writing performance, whether writing in one’s first language or L2 (Daly, 1978; Cheng, 2004; Limpo, 2018).

Further, students’ academic experiences shape their enjoyment of and anxiety about writing, which in turn informs their self-efficacy for writing (Martinez et al., 2011). The relationships between affect for writing, other motivational factors, and writing achievement have been mixed for undergraduate students writing in their L2. For example, Sabti et al. (2019) found that writing anxiety was unrelated to self-efficacy and writing achievement goals among Iraqi EFL students. In contrast, Zerey and Müjdeci (2023) reported that affect correlated positively. In contrast, anxiety correlated negatively to Turkish EFL undergraduates’ writing scores and their self-efficacy, adaptive goal orientations, and beliefs about writing. Similarly, Taiwanese EFL students’ second-language writing anxiety shared negative correlations with their self-efficacy for writing in English and their English writing grades (Cheng, 2004). Similar patterns were reported for Chinese undergraduates studying English online. Increased anxiety levels reduced Chinese students’ motivation to learn English and hampered their self-regulated English learning (Wang and Zhan, 2020). However, the relationship between English learners’ affect and writing in English has primarily been studied in their home countries or EFL contexts. However, studying internationally may add another layer of complexity due to the added cultural expectations for writing in the United States. Thus, international students’ affect for writing and its relation to writing in English is less well understood.

### Current study and research questions

The current study examines the dimensionality of international students’ self-efficacy, beliefs, goal orientation, and affect for writing and their contributions to performance in Academic English classes. In this study, international students completed surveys tapping their self-efficacy, motivational goals, beliefs, and affect for writing in English at the start and end of online, academic English writing courses. The courses’ final grades were used to measure students’ English writing achievement. Although prior research has explored the contributions of motivational factors to writing in English performance in their home countries (whether in United States schools or in EFL contexts), we had difficulty identifying literature examining the relationships among these factors among international students writing in English and studying in United States universities in an online context.

Further, there is limited consensus on the characterization of each motivational construct, which may reflect methodological, population, and contextual differences. First, studies have used principal component analysis (PCA; e.g., MacArthur et al., 2016) or exploratory factor analysis (EFA; Pintrich and Zusho, 2007; Ling et al., 2021) as means of reducing data and exploring self-efficacy, goal orientation, beliefs and affect for writing. Although both are powerful data reduction techniques, PCA is used to optimize the combination of variables into smaller subsets, or dimensions, whereas EFA is used to identify underlying constructs, or latent variables (Jain and Shandliya, 2013). Because the purpose of this paper was to replicate and extend findings from research examining the self-efficacy and beliefs constructs used for college-level writers (e.g., MacArthur et al., 2016), we first used PCA. However, it is unclear the extent to which PCA and EFA yield similar patterns for each motivational construct. Therefore, the current study seeks to explore the similarities and differences among the motivational constructs (self-efficacy, goal orientation, beliefs and affect for writing) revealed by EFA and PCA.

Furthermore, motivational constructs have been found to vary across populations and contexts. For example, Bruning et al. (2013) identified a 3-factor structure for self-efficacy among middle- and high-school students, whereas self-efficacy has been found to be unidimensional for United States college students (MacArthur et al., 2016; Ling et al., 2021). Studies involving largely domestic undergraduates in United States contexts have shown similar patterns for the motivational constructs for writing, both for students enrolled in developmental, or remedial, writing classes in a suburban community college (MacArthur et al., 2016), or a more diverse population of undergraduates enrolled in 4-year universities (Ling et al., 2021). It is also noteworthy that data collection for both studies took place before the COVID-19 pandemic when undergraduate education was primarily conducted in person. However, less is known about international students’ motivation for writing in English, as they navigate writing in their L2 in an educational system that differs not only in the language of instruction but also in its norms and practices from their secondary education in their home countries. Additionally, the transition to emergency remote instruction, when many international students studied online from their home countries, may have impacted students’ beliefs and attitudes toward their studies. Therefore, it was important for us to explore the components of the motivational constructs, their malleability, and their relationship to student performance. More specifically, this study focused on answering the following research questions:

1. In what ways do EFA and PCA reveal similarities and differences in the motivational constructs of self-efficacy, goal orientation, beliefs, and affect for writing among international students enrolled in online-academic English classes in a U.S. university?
2. To what extent does completing an Academic English course change international students’ self-efficacy, goal orientation, beliefs, and affect for writing in English?
3. To what extent do self-efficacy, goal orientation, beliefs and affect for writing explain international students’ performance in Academic English courses when instruction is provided online?

## Methods

### Study context and participants

This study occurred in a highly selective (less than 29% acceptance rate), large public research university in a suburban setting in the western United States. The campus is federally recognized as an Asian- and Hispanic-serving institution. Approximately 17% of undergraduate students are international, with 80 languages spoken and 87 countries represented within the undergraduate population.

All students enrolling in the university take the campus-developed analytic writing placement exam before their first term. The exam provides students with a prompt for writing an on-demand essay in 60 min. While students with scores above the threshold enroll in the lower-division composition courses, students below the threshold are counseled to take at least one of the Academic English courses. Academic English courses are offered at three different levels and are designed to prepare students for the lower-division composition courses required by all students. The first course covers academic language use and foundational academic writing skills such as summarizing and critiquing. In the second course, students organize and synthesize multiple sources and perspectives to develop an informed position on an academic topic. The third course provides students with practice in identifying, evaluating, analyzing, and presenting source information for credibility and relevance to an academic topic. The three Academic English courses have accompanying labs for further practice in academic writing.

We recruited eight instructors who were teaching 10 Academic English classes over a 10-week term in a quarter system (i.e., 10-week quarters rather than 15-week semesters). All international students in these classes were eligible for participation in this study. We collected surveys and grades for 98 students (44 female) enrolled in five classes (69 students enrolled in three level 2 classes and 29 students in two level 3 classes), taught synchronously online by five different instructors. One student was from the United States and was excluded from the analyses. Of the remaining 97 participants, 77 students took their courses internationally, 19 lived off-campus but in the United States, and one lived on campus. Most of the students were from China (84), with the remaining from Vietnam (4), Mexico (2), and one each from Cambodia, India, Japan, Kuwait, Myanmar, and Thailand, with one student responding with unrecognizable characters. Forty-seven students reported that this university was the first American school they had attended. Most students (94) were in their first year of studies, and three were in their second year. The study occurred in the spring of 2020 when all courses were taught remotely due to the pandemic.

### Measures

The measures included in this study included a demographic questionnaire, a motivation and self-efficacy questionnaire, and student grades in the course. The motivation and self-efficacy questionnaire was given twice, once in week 2 of the 10-week course and 8 weeks later at the end of the course (week 10).

#### Demographic questionnaire

During the second week of the term, students completed a survey to provide information about their home country, primary language, the language of instruction in school, age when they began learning English, their year of study at the university, gender, and frequency of using the campus writing center, as well as whether they had prior schooling in the United States. When reporting the age when English instruction began, some participants reported school grades. To this end, we treated “preschool” as age 3.5, “kindergarten” as age 5, and “first grade” as age 6. The responses “elementary school” (*n* = 1) and “middle school” (*n* = 1) were recoded as missing due to the broad range of grades covered. Participant demographics are summarized in Table 1.

**Table 1:** Participant characteristics and course achievement.

Variable	*n* (Total *n* = 97)
Gender	
Female	44
Male	53
Year of study at the university	
First year	94
Second year	3
Primary language	
Chinese (Mandarin, Cantonese)	84
Vietnamese	4
Arabic	1
Burmese	1
Japanese	1
Khmer	1
Spanish	1
Thai	1
Other	1
Language of instruction prior to university	
English	30
Chinese	55
Chinese and English	7
Japanese and English	1
Vietnamese	1
Vietnamese and English	1
Age when English instruction started (*N* = 79)	
Mean	7.11
Standard deviation	2.99
Range	3–16
Prior schooling in the United States	
Yes	47
No	50
Residence at time of study	
On campus	1
Off-campus, in the United States	19
International	77
Used the campus writing clinic before the course, M (SD)	0.27 (0.59)
Final grade, M (SD)	2.64 (1.04)
Passed course	
Passed	79
Failed	17

#### Motivation and self-efficacy questionnaire

A motivation and self-efficacy questionnaire was administered. This survey used a 5-point Likert scale (ranging from strongly disagree to strongly agree) and drew items from Bruning et al. (2013) and MacArthur et al. (2016). The self-efficacy scale contained 18 items and addressed students’ efficacy for different aspects of the writing process, such as organizing their ideas, evaluating and revising their writing, and writing different genres or parts of a paper. The achievement goal orientation scale consisted of three subscales. The first subscale, avoidance, included 4 items. The second subscale, performance, included three items, and the third, mastery, included 4 items. The third scale, the beliefs scale, included 6 items addressing students’ beliefs about the content and 6 items about writing conventions. The fourth scale contained 5 items that evaluated students’ affect about writing. Two items, *I do not like writing*, and *I avoid writing as much as possible,* were reverse-coded. For each item, we re-coded the Likert options as numbers where +2 was the strongest positive score, 0 was neutral, and − 2 was the strongest negative score. The score for each scale was the mean of its items, with +2 being the most positive and − 2 being the most negative.

#### Outcome variables

Student achievement was operationalized in two ways. First, students’ final grades in the writing course were recoded as a continuous variable using the university’s letter grade conversion policy (i.e., A+ = 4.0, A = 4.0, A− = 3.7, B+ = 3.3, B = 3.0, B− = 2.7, C+ = 2.3, C = 2.0, C− = 1.7, D+ = 1.3, D = 1.0, D− = 0.7, *F* = 0.0). Because two instructors only provided students’ pass/fail status, we could not convert their students’ scores into the continuous variable. Second, we created a binary student achievement variable characterized as “pass” or “fail.” For the second achievement variable, we created a score for all students who received letter grades using the university’s minimum passing score of C as the cut point.

### Procedures

During the second week of the term, the course instructors distributed an emailed recruitment for the study written by the second author. The recruitment included a hyperlink to the pretest survey that was administered using Qualtrics. The first screen of the survey was used to collect participants’ written informed consent to participate in this study. After providing written, informed consent, participants completed the demographic and motivational surveys. Eight weeks later, the motivational survey was administered once again through Qualtrics. After the courses ended, we retrieved final grades and pass/fail status from instructors.

### Analyses

All analyses were run using jamovi 2.3 (Jamovi Project, 2023). We first ran descriptive analysis for all the motivation survey items, with mean scores ranging between −0.62 and 1.58, and standard deviations ranging between 0.63 and 1.24 (Please see Supplementary Table S1). The values for skewness ranged between −1.53 and 0.52, and the values for kurtosis ranged from −0.88 to 1.81, which are within the cut-off values of |3.0| and |8.0|, respectively (Kline, 2011). Because less than 5% of the data were missing, as one participant was missing two variables, we deleted the missing case listwise from subsequent analyses (Baraldi and Enders, 2010).

To address the first research question, we conducted both an exploratory factor analysis (EFA) and a principal components analysis (PCA) within each of the motivational scales (self-efficacy, goal orientation, beliefs, and affect) specified by MacArthur et al. (2016) and Ling et al. (2021). Because of our relatively small sample, we calculated EFA using the principal axis (PA) method with Oblimin rotations on the pretest scores (Watkins, 2018). EFA factors were extracted based on parallel analyses. We also calculated PCA using Varimax rotations on the pretest scores to identify components based on parallel analysis (Tabachnick and Fidell, 2007). For each EFA and PCA, Bartlett’s test of sphericity <0.001 and the Kaiser-Meyer-Olkin measure of sampling adequacy >0.7, indicated that the assumptions for EFA and PCA were met (Watkins, 2018).

Next, we examined the extent to which online Academic English courses influence international students’ motivation for writing by calculating a 7 (motivation: self-efficacy, performance, mastery, avoidance, content, conventions, and affect) X 2 (time: pretest vs. post test) X 5 (class) repeated-measures multivariate analysis of variance (MANOVA), with motivation and time as repeated measures. We treated class as a between-subjects measure due to the nested data. Levene’s test indicated that the assumption of homogeneity of variance was not violated by any of the motivation variables. However, due to the nonsphericity of the motivation variables (Machauly’s *W* = 0.05, *p* < 0.001), we used the Greenhouse–Geisser correction. We used Scheffe *post hoc* tests to further examine significant main effects (note that all other assumptions for the MANOVA were met). Because repeated-measures MANOVA is an omnibus test, we calculated a series of repeated-measures ANOVAs on the pretest and post-test scores of each factor, using class as the between-subjects variable to address the nested nature of the data.

Finally, after running a correlation matrix to explore the relationships among the variables, we examined the contributions of the motivation factors on our two student achievement outcomes using hierarchical linear modeling (HLM). Hierarchical linear modeling is well-suited to the nested structure of our dataset, with students (level-1) clustered within classrooms (level-2; Raudenbush and Bryk, 2002). The hierarchical structure implies that students’ performance in their writing classes is influenced both by their individual characteristics and their class. After testing the assumptions of HLM, including linear relationships between each predictor variable and achievement outcomes, homogeneity of variance, and multivariate normality, we found that all assumptions were met.

For both models, level-1 variables were students’ ages when their English instruction began, gender, whether this was the first U.S. school the student attended, and prior use of the campus writing clinic, as well as the motivational variables at pretest (self-efficacy, performance orientation, mastery orientation, avoidance orientation, beliefs about content, beliefs about conventions, and affect for writing).

The first model was a logistic mixed model with a logit link for binary outcomes to predict the likelihood that a student passed the Academic English class. We report the fixed effects parameter estimates (β), odds ratio (OR), and the corresponding probability values (*p*). The modeling started with the null model (Model 0) to estimate the intraclass correlations (ICCs) and determine the proportion of variance accounted for by clustering within classes. We then fit Model 1 by adding all the level-1 student variables listed above.

For the second analysis, we used linear mixed model analysis to estimate students’ final grades. Because of our small sample, we used restricted maximum likelihood to reduce the bias that may occur with maximum likelihood estimation for small samples (Kenward and Roger, 1997). We first calculated the null model to estimate the ICCs. Next, we fit Model 1 with the same level-1 student variables included in the previous analysis.

## Results

### Dimensions of the motivation for writing scales for international students enrolled in an online academic English course

#### Self-efficacy

The results of the EFA and PCA for the self-efficacy scale are summarized in Table 2. The EFA revealed a 2-factor model, explaining 42% of the variance, that showed a marginally acceptable fit, with *χ*^2^ = 167, *df* = 118, *p* = 0.002, RMSEA = 0.07, 90% CI = (0.04, 0.09), TLI = 0.89. The latent constructs of self-efficacy for writing processes and self-efficacy for self-regulation were moderately correlated (*r* = 0.61).

**Table 2:** Self-efficacy for writing.

	Principal Axis EFA with oblimin rotation		PCA with varimax rotation
	Writing process	Self-regulation	Self-efficacy
I can write an essay with a strong conclusion	0.805		0.595
I can organize my ideas into a plan that makes sense	0.768		0.71
I can write a good persuasive essay	0.699		0.776
I can write paragraphs with details to support the main ideas	0.693		0.708
I can find the right words to express my ideas	0.639		0.74
I can think of good ideas to include in my writing when I am planning	0.612		0.637
I can write a paragraph that has a clear topic sentence	0.502		0.621
I can write an interesting introduction that makes the reader want to read the paper	0.464		0.6
I can evaluate whether my paper is well written	0.428		0.485
I can write a summary of the important points from an article I read	0.387	0.339	0.681
I can plan time to get my writing done by the deadline		0.778	0.656
I can edit my papers to fix errors		0.659	0.543
I can keep writing even when it’s difficult		0.611	0.581
I can focus on my writing for at least 1 h		0.605	0.534
I can revise my papers to make them better		0.561	0.745
I can evaluate whether I am making progress in learning to write		0.428	0.56
I can use a chart or graphic organizer to plan how to present my ideas		0.389	0.594
I can avoid distractions while I write		0.378	0.477
Eigenvalue	4.38	3.23	7.16
% of variance	24.4	18	39.8
Cronbach’s α	0.88	0.84	0.9

Although the PCA explained comparable (40%) variance, it was more consistent with the literature by extracting a single dimension for self-efficacy (MacArthur et al., 2016; Ling et al., 2021). The self-efficacy principal component had high reliability (Cronbach’s *α* = 0.90), and all 18 items had component loadings greater than 0.45.

#### Achievement goal orientation

Table 3 shows the results of the EFA and PCA for goal orientation. The findings of the EFA were consistent with the three-factor models of goal orientation reported in the literature (MacArthur et al., 2016; Ling et al., 2021). The three-factor model accounted for 65% of the variance. Although χ^2^ = 33.3, *df* = 25, *p* = 0.124, the other parameters indicated a good fit, RMSEA = 0.06, 90% CI = (0.00, 0.10), TLI = 0.97. Whereas performance and master orientations were moderately correlated (*r* = 0.61), neither were correlated with avoidance orientations (*r* = −0.06 and − 0.10, respectively).

**Table 3:** Goal orientation for writing.

	Principal Axis EFA with oblimin rotation: factors			PCA with varimax rotation: components	
	Performance	Mastery	Avoidance	Performance and mastery	Avoidance
I’m trying to complete all the assignments for the class	0.856			0.764	
I’m trying to get a good grade in the class	0.816			0.771	
I’m trying to pass this class	0.811			0.786	
I’m trying to become a better writer	0.535	0.413		0.876	
I’m trying to better organize my ideas		0.901		0.802	
I’m trying to improve how I express my ideas		0.887		0.837	
I’m trying to persuade others with my writing		0.705		0.618	
I’m trying to hide how nervous I am about writing			0.882		0.883
I’m trying to avoid making mistakes in front of my classmates			0.712		0.789
I’m trying to keep people from thinking I’m a poor writer			0.699		0.788
I’m trying to hide that I have a hard time writing			0.646		0.759
Eigenvalue	2.49	2.41	2.21	4.33	2.63
% of variance	22.7	21.9	20	39.4	23.9
Cronbach’s α	0.89	0.86	0.82	0.89	0.82

On the other hand, the PCA extracted two dimensions but similarly explained 64% of the variance. The seven items intended to measure performance goals and mastery orientations loaded onto a single component, explaining 40% of the variance. The four items intended to measure avoidance goals loaded onto the second component, explaining an additional 24% of the variance. The performance/mastery and avoidance components had high reliabilities with *α* = 0.89 and *α* = 0.82, respectively.

#### Beliefs

Table 4 shows that the PCA and EFA revealed similar patterns for students’ beliefs about writing that matched the 2-factor models in the literature (MacArthur et al., 2016; Ling et al., 2021). The 2-factor model explained almost half (47%) the variance. Although *χ*^2^ = 53.3, *df* = 43, *p* = 0.135, the other parameters indicated a good fit, RMSEA = 0.05, 90% CI = (0.00, 0.09), TLI = 0.96. The two factors, beliefs about content and beliefs about conventions, were uncorrelated, *r* = 0.18.

**Table 4:** Beliefs about writing – rotated component matrix.

	Principal axis EFA with oblimin rotation: factors		PCA with varimax rotation: components	
	Content	Conventions	Content	Conventions
Writing helps make my ideas clearer	0.879		0.873	
Writing helps me think about my topic in a new way	0.815		0.84	
I learn new things from writing	0.811		0.839	
Writing is one of the best ways to explore new ideas	0.7		0.763	
Revising helps me clarify my ideas	0.693		0.748	
Good writers discover new ideas while writing	0.602		0.682	
Good writers do not make errors in grammar		0.673		0.737
Good writers have to be able to write long complex sentences		0.65		0.719
Good writers need little revision because they get it right the first time		0.623		0.706
The main problem of poor writers is using incorrect grammar		0.586		0.681
Writing quickly is an important part of good writing		0.515		0.628
Revising is mostly about fixing errors in grammar and spelling		0.445		0.559
Eigenvalue	3.44	2.19	4.07	2.61
% of variance	28.7	18.3	33.9	21.8
Cronbach’s α	0.88	0.76	0.88	0.76

The two components extracted by the PCA were very similar to the EFA’s factors. These dimensions explained 56% of the variance. The six items intended to measure students’ beliefs about writing content loaded onto a single component, explaining 32% of the variance. The six items intended to measure students’ beliefs about the conventions of writing loaded onto the second component, explaining an additional 24% of the variance. The reliability was high for content beliefs (*α* = 0.88) and acceptable for conventions beliefs (*α* = 0.76).

#### Affect

The results of the EFA and the PCA for the affect scale are summarized in Table 5. The EFA yielded a 2-factor model of affect for writing, *χ*^2^ = 0.525, *df* = 1, *p* = 0.469, RMSEA = 0.00, 90% CI = (0.00, 0.24), TLI = 1.03. Together, the two factors, positive affect and negative affect, accounted for 63% of the variance and were moderately correlated, *r* = 0.56. In contrast, the PCA yielded a single component for affect, explaining 40% of the variance. The final affect component had high reliability (Cronbach’s *α* = 0.89).

**Table 5:** Affect for writing – rotated component matrix.

	Principal axis EFA with oblimin rotation: factors		PCA with varimax rotation: components
	Positive affect	Negative affect	Affect
The process of writing is satisfying for me	0.921		0.793
I think that writing is interesting	0.741		0.859
I usually enjoy writing	0.667		0.88
I do not like to write*		0.741	0.759
I try to avoid writing as much as possible*		0.63	0.519
Eigenvalue	1.98	1.17	2.99
% of variance	39.5	23.3	59.7
Cronbach’s α	0.87	0.63	0.89

Please note, items marked with * have been reverse-coded.

### Do online academic English courses affect international students’ motivation for writing?

Although the factors revealed by the EFAs were largely consistent with the literature (MacArthur et al., 2016; Ling et al., 2021), the models extracted generally had mediocre fits at best. Although these findings are suggestive of the underlying motivational factors, the exact weighting of each item is unclear. The PCA findings also did not exactly align with the EFA findings, sometimes identifying a different number of components (likely due to the purpose of reducing the overall amount of variance, rather than identifying constructs).

For these reasons, we constructed motivational variables that reflected the factors in the previous literature by calculating the mean of their constituent variables. Specifically, we calculated a single variable for self-efficacy using the mean of all items in the self-efficacy scale. For goal orientation, performance orientation was the mean of completing assignments passing the class, getting good grades, and becoming a better writer. Mastery orientation was the mean of becoming a better writer, improving at organizing ideas and expressing ideas, and persuading others. Avoidance orientation was the mean of the reverse-coded variables (hiding their nervousness, hiding that they are a poor writer and having a hard time writing, and avoiding making mistakes), so that positive scores would indicate less avoidance. Beliefs about content and conventions were the means of the variables shown in Table 4. When affect for writing was calculated by using the mean of all five variables, with disliking writing and avoiding writing being reverse coded so that positive scores would reflect more positive affect.

Table 6 summarizes students’ mean ratings for each motivational construct at the start and end of the writing course. Overall, students’ ratings across the motivation dimensions varied, *F* (3.22, 222.25) = 87.52, *p < 0*.001, *η^2^_p_* = 0.56. Scheffe *post hoc* tests revealed that overall, students’ performance goals were stronger than mastery goals, *t* (69) = 6.08, *p* < 0.001. Mastery goals received higher scores than their endorsed beliefs about content, *t* (69) = 3.20, *p* < 0.001, which was stronger than their self-efficacy for writing, *t* (69) =6.20, *p* <. 001. Students showed greater self-efficacy than affect for writing, *t* (69) =8.80, *p* < 0.001. However, affect for writing, beliefs about conventions, and avoidance orientations were comparable. Although the change in overall motivation scores was not significant, *F* (1,69) =0.61, *p = 0*.44, the interaction between the motivation constructs and pretest-posttest was significant, *F* (3.27,13.10) =3.84, *p = 0*.008, indicating that change across the motivational factors varied across the term. A series of Bonferroni-adjusted repeated measures ANOVAs found that at the end of the course, students showed increased self-efficacy, *F* (1,75) = 7.82, *p* < 0.001, *η^2^_p_* = 0.28 and improved affect for writing, *F* (1,74) = 5.93, *p* < 0.017, *η^2^_p_* = 0.07. Students also showed a decrease in their performance orientation, *F* (1,74) = 7.43, *p* < 0.008, *η^2^_p_* = 0.09. No other effects were significant.

**Table 6:** Means and standard deviations of student motivation scores at pretest and post test.

Motivation construct	Pretest	Post test
Self-efficacy: Mean (SD) Cronbach’s α	0.56 (0.51) 0.90	0.77 (0.48) 0.90
Goals – Performance Mean (SD) Cronbach’s α	1.52 (0.50) 0.89	1.40 (0.55) 0.86
Goals – Mastery Mean (SD) Cronbach’s α	1.26 (0.60) 0.86	1.19 (0.52) 0.82
Goals – Avoidance Mean (SD) Cronbach’s α	−0.10 (0.80) 0.82	−0.10 (0.88) 0.86
Beliefs – Content Mean (SD) Cronbach’s α	1.03 (0.67) 0.88	1.10 (0.55) 0.86
Beliefs – Conventions Mean (SD) Cronbach’s α	−0.24 (0.75) 0.76	−0.17 (0.84) 0.86
Affect Mean (SD) Cronbach’s α	0.05 (0.62) 0.82	0.18 (0.67) 0.81

### How do the motivational constructs contribute to international students’ performance in academic English courses?

The relations among the motivational constructs at the start of the term and with course outcomes are presented in Table 7. Two key patterns of association emerged among the motivational dimensions. First, self-efficacy, mastery orientations, content beliefs, and affect shared small to moderate positive correlations, with correlations ranging between *r* = 0.27 and *r* = 0.64. These correlations are consistent with the literature, suggesting the connections among self-efficacy, mastery goal orientations, beliefs about the involvement of expressing and exploring ideas in writing, and positive affect about writing. Although performance and mastery orientations were highly correlated (*r* = 0.70), as the literature suggests, mastery orientation was correlated with affect (*r = 0*.27) while performance orientation was not (*r* = 0.09) The second key correlation was a moderate, negative association between avoidance goals in writing and beliefs about writing conventions (*r* = −0.42). Students guided by avoidance goals were more likely to hold beliefs about the importance of the conventions in writing. However, student performance and the motivation factors shared only one bivariate correlation, which was between performance orientation and final grades, *r* = 0.29, *p* < 0.05.

**Table 7:** Correlations among the motivational constructs at the start of the term with course performance.

	Pretest motivational constructs							Student outcomes	
	Self-efficacy (*N* = 93)	Goals – performance (*N* = 93)	Goals – mastery (*N* = 93)	Goals – avoidance (*N* = 93)	Content (*N* = 94)	Conventions (*N* = 94)	Affect (*N* = 93)	Final grade (*N* = 52)	Pass (*N* = 93)
Self -Efficacy	--								
Goals - Performance	0.29**	--							
Goals - Mastery	0.54***	0.70***	--						
Goals - Avoidance	0.12	0.04	0.06	--					
Beliefs - Content	0.50***	0.42***	0.64***	−0.02	--				
Beliefs -Conventions	−0.09	−0.10	0.01	−0.42***	0.18	--			
Affect	0.47***	0.09	0.27**	0.19	0.55***	−0.01	--		
Final Grade	0.19	0.29*	0.22	0.14	0.07	−0.03	0.01	--	
Pass	0.11	0.16	0.13	0.11	−0.04	−0.06	0.05	0.85***	--

*p < 0.05; **p < 0.01; ***p < 0.001.

The logistic mixed model provides information on the likelihood of students passing the Academic English class (see Table 8). The null model revealed that the ICC was 0.56, indicating that half the variance could be attributed to differences between the classes. Interpreting our data at the student level, only one motivation factor predicted whether students passed the Academic English class. Students with greater beliefs about content were associated with lower passing rates, *OR* = 0.07, *p* = 0.034. That is, students who held stronger beliefs about the role of expressing and exploring ideas in writing were less likely to pass the Academic English course.

**Table 8:** Logistic mixed model of the contributions of motivation factors at pretest to passing academic English classes.

Parameter	Model 0			Model 1		
	β (SE)	Odds ratio	*p*	β (SE)	Odds ratio	*p*
Fixed effects						
Intercept	1.65 (0.95)	5.21	0.10	2.41 (1.39)	11.12	0.08
*Student Predictors*						
Age English learned				0.03 (0.17)	1.03	0.85
Gender (female v. male)				1.21 (1.06)	3.36	0.25
First United States school				−0.63 (0.92)	0.53	0.49
Prior writing clinic use				1.12 (1.29)	3.05	0.39
Self-efficacy				0.15 (1.39)	1.16	0.91
Performance				2.07 (1.21)	7.90	0.09
Mastery				1.16 (1.17)	3.18	0.32
Avoidance				0.42 (0.85)	1.52	0.63
Beliefs – Content				−2.68 (1.43)*	0.07*	0.05*
Beliefs – Conventions				0.10 (0.79)	0.79	0.90
Affect				1.41 (1.38)	4.09	0.30
Random Effects						
Classroom (SD)	1.93			2.09		
ICC	0.56			0.67		
Total *R*^2^				0.76		

The results of the hierarchical linear model predicting final grades in the Academic English courses are presented in Table 9. The ICC of the null model was 0.43, indicating that substantial variance (43%) in students’ final grades could be attributed to differences between the classes. When student-level variables were included in the model, over half the variance (58%) in students’ final grades was accounted for. Although student demographic variables did not account for students’ final grades, only one motivational construct predicted students’ final grades. Student self-efficacy at the start of the course was a unique, positive predictor of students’ final grades (*B* = 1.07, *p* = 0.003), indicating that an increase of one point on the self-efficacy scale was associated with an increase of 1.07 on the final grade, or an increase of a full letter grade (e.g., B to A). Beliefs about content trended as a unique, negative predictor of students’ final grades (*B* = −0.5, *p* = 0.08), suggesting a decrease in letter grades of almost two steps (e.g., A to B+) with each increased point on the contents belief scale.

**Table 9:** Hierarchical linear model of motivational factors at pretest predicting the final grades in academic English classes.

	Model 0		Model 1	
	β	SE	*β*	SE
Fixed Effects				
Intercept	2.57**	0.40	2.62**	0.39
*Student Predictors*				
Age English learned			−0.02	0.05
Gender (female v. male)			0.03	0.25
First American school (yes v. no)			−0.40	0.25
Prior use of the Writing Clinic			0.05	0.20
Self-efficacy (pretest)			1.07**	0.33**
Performance orientation (pretest)			0.52	0.29
Mastery orientation (pretest)			0.00	0.31
Avoidance orientation (pretest)			0.22	0.19
Beliefs - Content (pretest)			−0.52	0.28
Beliefs - Conventions (pretest)			0.30	0.20
Affect for writing (pretest)			−0.44	0.30
Random effects				
Classroom (SD)	0.77		0.74	
ICC	0.43		0.48	
Total R^2^			0.58	

*p < 0.05; **p < 0.01; ***p < 0.001.

## Discussion

The current study sought to characterize international students’ motivation for writing and its contribution to achievement in online academic English classes during the COVID-19 pandemic. More specifically, this study examined international students’ self-efficacy, goal orientation, beliefs and affect for writing, their malleability, and their contributions to writing achievement in academic English classes.

Our analyses of the four motivational constructs among international students taking online writing courses highlight the importance of understanding the methodologies, population studied, and context when attempting to characterize self-efficacy, goal orientation, beliefs, and affect for writing. Overall, the models extracted by EFA tended to have mediocre fits at best, whereas PCA was more successful in reducing the data into components. Further, only one construct, beliefs about writing, yielded matching 2-factor models (beliefs about content and conventions) that were consistent with the literature (MacArthur et al., 2016; Ling et al., 2021). Otherwise, we found that EFA tended to yield more complex structures than PCA.

When considering self-efficacy, the PCA was consistent with the literature (MacArthur et al., 2016; Ling et al., 2021) by reducing the data to a single dimension. In contrast, the EFA’s findings were similar to those of Pajares (2007), who reported a 2-factor model. However, the latent factors extracted with international undergraduates reflected different constructs than those revealed with K-12 students. Whereas the 2-factor model with K-12 students reflected students’ confidence in their developing skills in creating content and mastery of conventions (Pajares, 2007), for international students, the two-factors reflected more mature writing, self-efficacy for skills directly tied to writing (e.g., ideation, writing different genres or parts of papers, and planning) and self-efficacy for regulating the writing process (e.g., staying on task, meeting deadlines, and using tools such as graphic organizers to support writing). Thus, for undergraduates studying online to improve their writing in their L2, self-efficacy for the writing processes may be distinct from self-efficacy for managing their studies. Because this study took place early in the COVID-19 pandemic, when online instruction was more novel and most (80%) of the students were in their home countries, we encourage more research to better understand self-efficacy and motivation for the more typical international student experience with face-to-face instruction on campus.

The two other motivational constructs also yielded divergent findings. For goal orientation, the EFA yielded results like the three-factor models reported in the literature among monolingual students (Dweck, 1986; Elliot and Church, 1997; MacArthur et al., 2016; Ling et al., 2021), and EFL undergraduates (e.g., Farsani et al., 2014; Chea and Shumow, 2017; Sabti et al., 2019). In contrast, the PCA yielded two components, with the first component including the same items that loaded onto the mastery and performance orientation factors of the EFA, and the second component matching the EFA’s avoidance factor. Similarly, the affect for writing scale yielded divergent findings for EFA and PCA. Whereas the EFA revealed two factors (positive affect and negative affect), PCA revealed a single component that was consistent with the unitary construct reported in the literature (Ling et al., 2021). Taken together, these findings highlight the importance of considering the data reduction technique used. Although there was tremendous overlap in the sets of variables combined, EFA produced more complex models.

The correlations among the motivational dimensions were consistent with the literature. Self-efficacy, performance, and mastery orientations, beliefs about content, and affect shared moderate positive correlations. Like Ling et al. (2021), we found that affect for writing was correlated with mastery goal orientations, but not performance goals. Taken together, these correlations suggest that international students who are more confident in their writing skills tend to enjoy writing, focus on both mastering and attaining recognition for their writing and believe that good writing involves the exploration and development of ideas. Similar patterns of correlation have been reported with general populations of undergraduates (e.g., Ling et al., 2021), undergraduates in remedial writing programs (MacArthur et al., 2016), and students writing in their L2 in EFL contexts (Zerey and Müjdeci, 2023). The interrelationships among self-efficacy, goal orientation, beliefs about content, and affect have been thought to contribute to students’ use of self-regulated strategies and persistence in writing, leading to more favorable academic outcomes (Phakiti et al., 2013).

Conversely, our study highlighted a low, negative correlation between avoidance orientation and beliefs about writing conventions. Please recall, items on the avoidance scale were reversed-coded, so that lower scores were associated more strongly with the maladaptive goal orientation of avoidance. Thus, this correlation suggests that international students who were most concerned about concealing their perceived struggles in writing were more likely to believe that good writing is defined by spelling and grammatical conventions. Considering that these international students were placed in developmental, Academic English courses to prepare them for the general freshman composition courses, students’ desires to avoid appearing incompetent in writing in their L2 may reflect their need to acquire greater mastery of the writing conventions of English. Because we only had access to students’ final grades, it is unclear whether students’ beliefs about conventions reflect their mastery of the L2 writing conventions. Thus, future research might also examine writing samples to determine how international students’ beliefs about writing align with their performance. This work may be longitudinal, so that one may determine if international students’ beliefs about writing and avoidance goals change to reflect growing competence in their L2.

Our study indicated that some motivation dimensions, such as self-efficacy and enjoyment of writing, were malleable within the duration of a 10-week course. International students demonstrated increased self-efficacy and reported enjoying writing more by the end of the academic English courses, suggesting the potential for positive changes in motivation over time. Further, they reported lower performance orientations at the end of the course. The improved confidence and affect for writing at the end of the academic English courses are encouraging and serve to counter concerns that such classes may exacerbate low self-efficacy and anxiety and impede international students’ academic success (Pappamihiel, 2001; Yeh and Inose, 2003; Moss et al., 2014).

Finally, our study confirmed that motivation does contribute to writing performance, but the relationships were not always as anticipated. The first finding, that self-efficacy at the start of the course predicted students’ final grades, is unsurprising. Much of the literature reports self-efficacy to be a robust contributor to writing achievement for undergraduates in their L1 (Zimmerman and Bandura, 1994; Prat-Sala and Redford, 2012) and L2 (Phakiti et al., 2013; Chea and Shumow, 2017; Sabti et al., 2019). The second finding was more surprising, as content beliefs (beliefs that writing is about exploring and expressing ideas) contributed to slightly lower odds of passing the academic English writing classes. This paradox might lie in the cultural underpinnings of writing, which go beyond vocabulary and conventions and incorporate specific discourse norms. Writing as a cultural practice is susceptible to the influence of different discourse norms. Students who have always been high achievers might find it challenging to adapt to these new conventions while they continue to develop their L2 writing skills. This struggle could be more pronounced for students who perceive writing as a primary tool for exploring and expressing ideas (Durkin, 2008; Lee and Deakin, 2016; Heng, 2018) and who might be inadvertently adhering to their L1 rhetorical styles (Connor, 2004; Saffari et al., 2017; Wei et al., 2020).

One important limitation is that our findings are based on students’ outcomes in the academic English courses rather than their performance on the individual writing assignments. Without access to individual writing assignments, we could not explore how international students engaged in the writing process and communicated their ideas, limiting our ability to determine if these rhetorical differences were responsible for this relationship. However, MacArthur et al. (2016) found that beliefs about content shared negative correlations with writing performance among undergraduates taking remedial writing classes. Thus, the negative contributions may suggest that students in general who value writing for expressing ideas may be more common among undergraduates still developing their academic writing skills in English. Nonetheless, our findings support the need for explicit instruction in the rhetorical norms and styles of argumentation of their L2 for international students, particularly those who heavily value writing for the exploration and expression of ideas. Future studies could explore the relationship between international students’ beliefs about writing and their adoption of Western argumentation conventions.

The generalizability of these findings is also limited to some degree by the population and context of this study. That is, this study was conducted with international students taking these courses online during the COVID-19 pandemic. The population was made up of a majority (85.7%) students from China, which is not representative of the larger international student community. For example, students from China may differ from other international students in their self-efficacy for writing in English than students from countries with alphabetic written languages that may have more similarities to English. They may also hold different beliefs about what is important in writing than students from other countries, which may have impacted the findings. The COVID-19 pandemic may also have raised students’ anxiety levels or impacted their self-efficacy for writing or participation in university writing courses online. Future research should expand this work to additional populations of international students.

In conclusion, motivation for writing is multidimensional and contributes to international students’ success in academic English courses. With the rising number of international students attending English-speaking universities (Institute of International Education, 2022), universities have sought to help them develop the academic writing skills in English needed to succeed in their courses, increasingly through online course delivery (Kung, 2017). Remedial ESL or academic English courses may be an important way of supporting international students’ experiences in higher education not only by promoting the academic writing skills critical for academic success but also by building their self-efficacy and enjoyment of writing in English. Yet international students’ initial motivations and beliefs about writing may contribute to their success in these courses. Although their self-efficacy at the onset of the academic English courses was adaptive and contributed to students’ success, holding strong beliefs about the value of writing for exploring and expressing ideas contributed to poorer performance. Thus, instructors may wish to be particularly attentive to international students’ initial beliefs about writing, so they might adapt instruction to clarify misconceptions about effective academic writing in English. Our study underscores the need for a more nuanced understanding of the different motivational dimensions, especially in a diverse linguistic and cultural context, and suggests potential avenues for pedagogical interventions to foster international students’ academic success.

## Data availability statement

The raw data supporting the conclusions of this article will be made available by the authors, without undue reservation.

## Ethics statement

Ethical review and approval was not required for the study on human participants in accordance with the local legislation and institutional requirements. Written informed consent for participation was not required for this study in accordance with United States federal legislation and institutional requirements.

## Author contributions

PC was involved in study design, analyzes and writing. ML was involved in study design and data collection. ME was involved in data analysis and writing the manuscript. MH was involved in data analysis and writing the manuscript. JL was involved in data cleaning and analysis. JWL was involved in study design and data collection. All authors contributed to the article and approved the submitted version.
