# Supplementary material for: The motivational beliefs and attitudes about writing of international students enrolled in online academic English classes during the COVID-19 pandemic
Source: Front Psychol. 2024 Jun 13;14:1232664. doi: 10.3389/fpsyg.2023.1232664 (PMC11208714; doi:10.3389/fpsyg.2023.1232664)
Supplement: Supplementary file 1 [file Table_1.DOCX]

Supplemental Table 1. Descriptive Statistics for Survey Items

| Item | Mean | S.D. | Skewness | Kurtosis |
| --- | --- | --- | --- | --- |
| Self-Efficacy | | | | |
| I can write a good persuasive essay | 0.34 | 0.79 | -0.55 | 0.41 |
| I can revise my papers to make them better | 0.53 | 0.77 | -0.23 | -0.30 |
| I can find the right words to express my ideas | 0.47 | 0.82 | -0.38 | 0.05 |
| I can organize my ideas into a plan that makes sense | 0.63 | 0.76 | -0.76 | 1.02 |
| I can write paragraphs with details to support the main ideas | 0.80 | 0.72 | -0.71 | 1.76 |
| I can write a summary of the important points from an article I read | 0.88 | 0.71 | -0.55 | 0.63 |
| I can plan time to get my writing done by the deadline | 1.04 | 0.76 | -0.52 | 0.12 |
| I can think of good ideas to include in my writing when I am planning | 0.67 | 0.75 | -0.62 | 1.09 |
| I can write a paragraph that has a clear topic sentence | 0.70 | 0.75 | -0.23 | -0.13 |
| I can write an interesting introduction that makes the reader want to read the paper | 0.20 | 0.75 | 0.42 | 0.97 |
| I can write an essay with a strong conclusion | 0.34 | 0.848 | -0.10 | -0.20 |
| I can use a chart or graphic organizer to plan how to present my ideas | 0.21 | 1.05 | -0.21 | -0.61 |
| I can keep writing even when it’s difficult | 0.51 | 0.87 | -0.51 | 0.30 |
| I can evaluate whether I am making progress in learning to write | 0.80 | 0.78 | -0.73 | 1.24 |
| I can edit my papers to fix errors | 0.68 | 0.79 | -0.30 | -0.21 |
| I can focus on my writing for at least 1 hour | 0.72 | 0.98 | -0.67 | 0.07 |
| I can evaluate whether my paper is well-written | 0.33 | 0.79 | 0.00 | 0.20 |
| I can avoid distractions while I write | 0.23 | 0.99 | -0.28 | -0.50 |
| Goal Orientation | | | | |
| I’m trying to hide how nervous I am about writing * | 0.15 | 1.03 | 0.17 | 0.62 |
| I’m trying to avoid making mistakes in front of my classmates* | -0.62 | 0.99 | 0.98 | 0.84 |
| I’m trying to keep people from thinking I’m a poor writer * | -0.09 | 1.01 | 0.38 | 0.52 |
| I’m trying to hide that I have a hard time writing* | 0.17 | 0.95 | -0.20 | 0.29 |
| I’m trying to become a better writer | 1.48 | 0.68 | -1.17 | 0.97 |
| I’m trying to improve how I express my ideas | 1.34 | 0.63 | -0.67 | 0.81 |
| I’m trying to better organize my ideas | 1.32 | 0.64 | -0.64 | 0.81 |
| I’m trying to pass this class | 1.58 | 0.69 | -1.56 | 1.73 |
| I’m trying to get a good grade in the class | 1.47 | 0.70 | -1.16 | 0.79 |
| I’m trying to complete all the assignments for the class | 1.56 | 0.66 | -1.44 | 1.75 |
| I’m trying to persuade others with my writing | 0.88 | 0.86 | -0.39 | 0.01 |
| Beliefs About Writing | | | | |
| Writing helps make my ideas clearer | 1.06 | 0.82 | -1.17 | 2.69 |
| Writing helps me think about my topic in a new way | 0.84 | 0.92 | -0.62 | 0.50 |
| I learn new things from writing | 1.02 | 0.90 | -1.03 | 1.44 |
| Writing is one of the best ways to explore new ideas | 0.88 | 0.91 | -0.72 | 0.71 |
| Revising helps me clarify my ideas | 1.18 | 0.71 | -0.64 | 0.43 |
| Good writers discover new ideas while writing | 1.19 | 0.75 | -0.48 | -0.54 |
| Good writers do not make errors in grammar | -0.27 | 1.22 | 0.400 | -0.85 |
| Good writers have to be able to write long complex sentences | 0.00 | 1.10 | -0.24 | -0.83 |
| Good writers need little revision because they get it right the first time | -0.49 | 1.24 | 0.54 | -0.71 |
| The main problem of poor writers is using incorrect grammar | -0.25 | 1.00 | 0.40 | -0.32 |
| Writing quickly is an important part of good writing | -0.36 | 1.01 | 0.46 | -0.10 |
| Revising is mostly about fixing errors in grammar and spelling | -0.15 | 1.05 | 0.19 | -0.80 |
| Affect for Writing | | | | |
| I usually enjoy writing | -0.04 | 0.82 | -0.15 | 0.79 |
| I think that writing is interesting | 0.18 | 0.77 | 0.10 | 0.26 |
| The process of writing is satisfying for me | 0.11 | 0.77 | 0.11 | 0.97 |
| I don’t like to write* | -0.09 | 0.90 | 0.01 | 0.24 |
| I try to avoid writing as much as possible* | 0.12 | 0.85 | 0.31 | -0.05 |

* Items have been reverse-coded
